# Smith-Lemli-Opitz Syndrome: A Case with Annular Pancreas

**DOI:** 10.1155/2014/623926

**Published:** 2014-08-05

**Authors:** Mehmet Demirdöven, Hamza Yazgan, Mevlit Korkmaz, Arzu Gebeşçe, Alparslan Tonbul

**Affiliations:** ^1^Department Of Pediatrics, School Of Medicine, Fatih University, Turkey; ^2^Fatih Üniversitesi Tıp Fakültesi, Sahil Yolu Sokak No. 16 Dragos Maltepe, 34844 Istanbul, Turkey; ^3^Department Of Pediatrics Surgery, School Of Medicine, Fatih University, Turkey

## Abstract

Smith-Lemli-Opitz syndrome is an autosomal recessive disease of cholesterol metabolism. It is a multiple malformation syndrome with typical dysmorphic features such as bitemporal narrowing, ptosis, epicanthus, microcephaly, micrognathia, and cardiovascular, skeletal, urogenital, and gastrointestinal anomalies. This report presents a typical case of Smith-Lemli-Opitz syndrome with annular pancreas which is an unreported gastrointestinal abnormality.

## 1. Introduction

Smith-Lemli-Opitz syndrome (SLOS) is an autosomal recessive disease caused by an inborn error of cholesterol metabolism due to the deficiency of the enzyme 7-dehydrocholesterol reductase. It is by far the most common disorder of the postsqualene cholesterol biosynthesis. The incidence is reported to be approximately 1/20 000-1/70 000 and is more common in people of European descent [1]. The clinical phenotype ranges from mild to severe with classic SLOS patients having characteristic faces that include microcephaly, ptosis, anteverted nares, and micrognathia; growth and mental retardation; hypogenitalism in males; and skeletal abnormalities, the most common being 2,3 toe syndactyly and postaxial polydactyly. Many gastrointestinal abnormalities were also reported in SLOS patients including colonic aganglionosis, cholestatic liver disease, and pyloric stenosis [2].

Herein we report a case of SLOS with annular pancreas resulting in duodenal obstruction that is not reported so far.

## 2. Case

**Figure 1:**
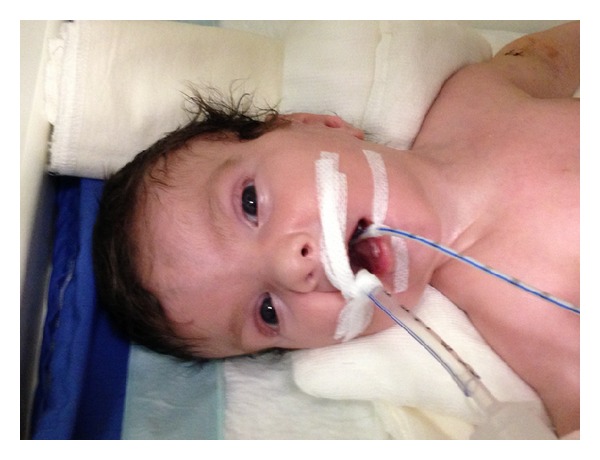
Typical dysmorphic face of the patient.

**Figure 2:**
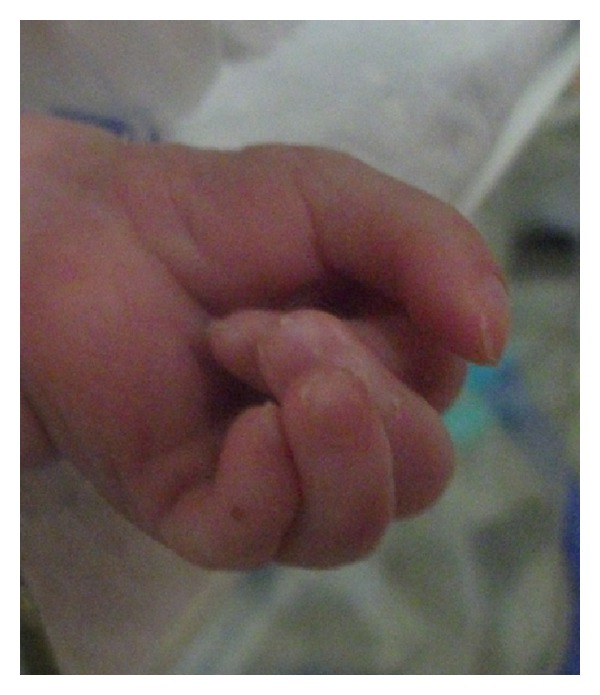
Overriding fingers.

**Figure 3:**
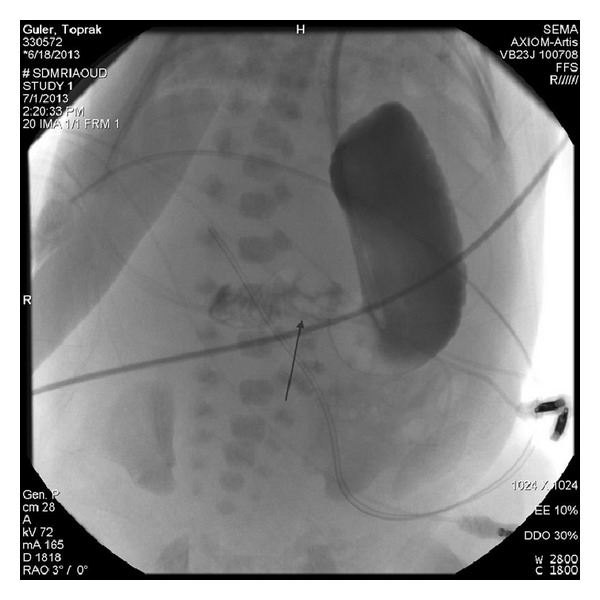
Upper GI series showing difficulty in gastric emptying and insufficient distention at the pylorus and duodenum (arrow) due to the encircled pancreatic tissue.

The patient was a male baby born at 34 gw from 24-year-old primigravid mother by spontaneous vaginal delivery. There was a first degree consanguinity between parents. The physical examination revealed a 3200 gr (>90 p), 48 (75–90 p) cm hypotonic, cyanotic baby with hydrops fetalis. Head circumference was 35 cm (>90 p). Bitemporal narrowing, ptosis, cataracts, short nasal root, anteverted nares, micrognathia, low-set ears, overriding fingers, and scrotal hypoplasia with cryptorchidism were noted (Figures 1 and 2). There was a 2/6 grade systolic mesocardiac murmur. Abdominal distention was also remarkable with 4 cm hepatic and 2 cm splenic enlargement. Ultrasonographic evaluation of abdomen demonstrated right ptotic kidney and grade 2 pelvicaliceal ectasia in left kidney. Echocardiography revealed VSD, ASD, PDA, and pericardial effusion. Cranial ultrasound was normal. Because of the typical dysmorphic features, SLOS was the clinical diagnosis. Serum cholesterol and dehydrocholesterol levels were 74 mg/dL and 4.2 *μ*g/mL, respectively. 7-Dehydrocholesterol (DHC7)/cholesterol ratio was 0.56 (normal: 0.16 ± 0.09 *μ*g/mL). Basal serum cortisol level was 32 *μ*g/dL. During followup in NICU the patient had feeding intolerance with bile stained vomitus. Radiologic evaluation of gastrointestinal system delineated delayed passage from pylorus, a filling defect at the base of the duodenal bulb and also a filling defect at the antral region that is consistent with a web. At the surgical exploration, stomach and the first part of the duodenum were dilated and there was an obstruction at the second part of the duodenum caused by annular pancreas (Figure 3). After operation, his condition was deteriorated and he died of respiratory failure 37 days after birth.

## 3. Discussion

Cholesterol is an important lipid molecule that is essential for cellular membrane for proper membrane permeability and fluidity. It is also a precursor for the synthesis of steroid hormones, bile acids, and vitamin D. Cholesterol also functions in intracellular transport, cell signaling, and nerve conduction.

Abnormalities in cholesterol biosynthesis result in some of the several dysmorphology syndromes, namely, desmosterolosis, lathosterolosis, CHILD syndrome (a form of chondrodysplasia punctata), Greenberg dysplasia, and the more common SLOS [3]. The pathogenesis of these multisystem malformations has not been clear yet. Some of them have been attributed to impaired sonic hedgehog (SHH) functioning which plays a key regulatory role in vertebrate organogenesis, such as in the growth of digits and organization of the brain [4, 5].

SLOS is a multiple congenital malformation syndrome caused by deficiency of the enzyme 7-dehydrocholesterol reductase encoded by DHCR7 gene located on chromosome 11q13.4. The diagnosis requires measurement of serum cholesterol and 7-dehydrocholesterol levels. Although most affected individuals have hypocholesterolemia, there may be an overlap in cholesterol levels of normal and affected individuals especially when the affected individuals are older or have a milder phenotype. Age-appropriate values must be considered for diagnosis (85–165 mg/dL in neonates) [6]. Serum cholesterol was low (74 mg/dL) in our patient. Normal range for 7DHC is 0.16 ± 0.09 *μ*g/mL [7]. In our patient, the level of 7DHC was high (4.2 *μ*g/mL) and the ratio was 0.56 confirming the diagnosis of SLOS.

Clinical characteristics include mental retardation, developmental delay, failure to thrive, autism, photosensitivity, microcephaly, bitemporal narrowing, broad nose with anteverted nares, micrognathia, ptosis, epichantal folds, cataracts, optic nerve hypoplasia, ASD, VSD, PDA, pyloric stenosis, Hirschsprung disease, hypospadias, cryptorchidism, renal anomalies, and skeletal abnormalities like rhizomelia, 2-3 toe syndactyly, and polydactyly [8].

Infants with SLOS frequently have feeding problems secondary to a combination of hypotonia, oral-motor incoordination, cleft palate, dysmotility, hypomotility, gastrointestinal reflux, constipation, and formula intolerance [9]. Hirschsprung disease and hypertrophic pyloric stenosis have been described [10, 11]. The frequency of hepatic manifestation is reported to be 2.5–16% [12]. Cholestatic liver disease and isolated hypertransaminasemia are among them. In addition to typical dysmorphic features of SLOS, our patient had duodenal obstruction caused by annular pancreas that has not been described so far.
